# The correlation between neutrophil-to-lymphocyte ratio, carcinoembryonic antigen, and carbohydrate antigen 153 levels with chemotherapy-related cognitive impairment in early-stage breast cancer patients

**DOI:** 10.3389/fmed.2022.945433

**Published:** 2022-08-25

**Authors:** Sheng Yu, Jingjing Zhao, Menglian Wang, Guo Cheng, Wen Li, Lingxue Tang, Senbang Yao, Lulian Pang, Xiangxiang Yin, Yanyan Jing, Huaidong Cheng

**Affiliations:** ^1^Department of Oncology, The Second Affiliated Hospital of Anhui Medical University, Hefei, China; ^2^Department of Finance, University of Connecticut, Storrs, CT, United States

**Keywords:** inflammatory biomarkers, adverse effects of chemotherapy, cognitive evaluation and diagnosis, management of breast cancer, neutrophil-to-lymphocyte ratio, carbohydrate antigen 153

## Abstract

**Background:**

*The changes in inflammation and tumor biomarkers are associated with the anti-tumor immunological processes.* Early detection and intervention are of great significance to the clinical management of cancer-related diseases. Peripheral blood biomarkers [e.g., neutrophil-to-lymphocyte ratio (NLR), carcinoembryonic antigen (CEA), and carbohydrate antigen 153 (CA153)] are obtained in real-timely, conveniently, and less invasively, and proved to availably predicted the disease states and prognosis of various cancers, including breast cancer (BC). Inflammation and poor disease management promote cognitive impairment. Chemotherapy-related cognitive impairment (CRCI) hazard long-term survival and quality of life (QOL) of BC patients, but its correlation with NLR, CEA, and CA153 is not clear.

**Purpose:**

This study aimed to investigate changes in NLR, CEA, and CA153 levels before and after chemotherapy and their correlation with CRCI in patients with early-stage BC.

**Materials and methods:**

The 187 patients with BC who were measured for NLR, CEA, and CA153 values within the first 24 hours of admission, were assigned into two groups: the before/after chemotherapy group (BCG/ACG). The ACG was assigned into two subgroups based on the cognitive assessment results: the cognitive normal/impaired group (CNG/CIG). Patients’ self-perceived cognitive impairments were evaluated using a mini-mental state examination (MMSE), prospective and retrospective memory (PM and RM) questionnaire (PRMQ), and functional assessment of cancer therapy-cognitive function version 3 (FACT-Cog, version 3, including CogPCI, CogOth, CogPCA, and CogQOL). Their QOL was also evaluated.

**Results:**

The NLR and CA153 levels were elevated after chemotherapy (BCG vs ACG: *Z* = −1.996 and −1.615, *P* = 0.046 and 0.106, respectively), and significantly elevated in patients with CRCI (BCG vs CIG: *Z* = −2.444 and -2.293, *P* = 0.015 and 0.022; respectively). However, there was not reach significant difference in CEA levels between the four groups. In addition, there was a weak to moderate correlation between peripheral blood biomarkers (NLR, CEA, and CA153) levels and CRCI (*r* = −0.404, −0.205, −0.322; respectively; *P* < 0.001). Cognitive impairment scores (MMSE, PM, RM, and FACT-Cog) had a strong correlation with QOL in patients with early-stage BC (*r* = −0.786, 0.851, 0.849, and 0.938; respectively; *P* < 0.001).

**Conclusion:**

NLR and CA153 m be valuable diagnostic adjuncts of CRCI, and CRCI has a strong correlation with QOL in patients with early-stage BC.

## Introduction

Breast cancer (BC) seriously threatens women’s health with 2.26 million new cases and ranked first worldwide in 2020 (1). Chemotherapy, hormone, and targeted therapy are critical treatments for BC, and chemotherapy is essential for neoadjuvant/adjuvant therapy for early-stage patients with BC (2, 3). Chemotherapy improves the survival time of patients with BC but impairs cognitive function. A meta-analysis showed that doxorubicin, cyclophosphamide, epirubicin, 5-fluorouracil, and taxane induced different degrees of cognitive impairment (4). Prevalence statistics of chemotherapy-related cognitive impairment (CRCI) among patients with BC are inconsistent, from 16 to 75%, according to criteria *via* quantified impairment (5). CRCI can persist for years after chemotherapy (6, 7), deeply understanding and better management of CRCI are significant for the long-term survival and quality of life (QOL) of patients with BC.

Chemotherapy-related cognitive impairment seriously decreases QOL. CRCI increases patients’ difficulty in integrating into society *via* impairing memory, executive function, processing speed, and attention (8). Young cancer survivors are more likely to be disturbed by cancer in their family life and social activities. Young women are more prone to fatigue, constipation, gastrointestinal symptoms, lymphedema, poor body image, impaired sexual ability, and unemployment than older women (9). Even minor cognitive impairment will seriously reduce patients’ social participation and employability, leading to a decline in confidence and QOL (10). In particular, patients with BC are often young with long-term survival and who desire to return to their normal life and work after treatment (11). Therefore, CRCI may be a major obstacle due to it hinders most aspects of daily life and QOL. CRCI results from multifactorial, and the specific mechanism remains unclear. Antineoplastic drugs, genetic susceptibility, inflammation, and psychological disorders such as fatigue and depression are possible risk factors (12). Chemotherapy caused CRCI *via* various mechanisms, including DNA damage, and inflammation, impairing the permeability and integrity of the blood-brain barrier (BBB), and promoting endocrine and metabolic disorders (13). Researchers diagnosed CRCI *via* various methods, including self-reported questionnaires, functional magnetic resonance imaging (FMRI), and neuropsychological tests (14). These methods require improvement due to the demands for patient cooperation, unconcealment, and high expense. Blood routine is well revealed the inflammatory level of the body. It is vital to detect early and potential CRCI patients *via* real-time and convenient methods.

Peripheral blood is obtained less invasively to assess the patient’s disease status within the first day of admission. The inflammation levels associate with mental disorders and are assessed by peripheral blood (15). Ning et al. showed that inflammation promoted peripheral immunity and CRCI by regulating the expression of brain-derived neurotrophic factor (BDNF) (16). Our previous study found that the levels of peripheral inflammation (interleukin-1β (IL-1β), tumor necrosis factor-α (TNF-α), and IL-4) were closely related to CRCI in patients with BC (17). Inflammation promoted cancer progression by stimulating angiogenesis and lymphangiogenesis and reshaping the immune microenvironment (18). The neutrophil-to-lymphocyte ratio (NLR) is an inexpensive and simple inflammatory marker, and its high levels indicated a poor prognosis in many solid tumors (19). Ahmed et al. showed that high NLR levels predisposed to mental illness after COVID-19 (20). Yong et al. showed that preoperative NLR levels were a risk factor for cognitive impairment after radical gastrectomy in elderly patients (21).

Elevated levels of inflammation and tumor biomarkers indicate poor cancer control. For example, high carcinoembryonic antigen (CEA) levels were correlated with increased inflammation during treatment with oxaliplatin in colorectal cancer (22). Tumour biomarker increase followed by whole-body imaging is highly effective for early detection and localization of tumor recurrence in clinically asymptomatic patients with BC (23). CEA levels were elevated in multiple diseases, including 49 cancers (e.g., colon, breast, and lung cancer) and non-cancer diseases (e.g., lung fibrosis) (24). The increase of CEA in colon adenocarcinoma patients after surgery indicates a high risk of recurrence (25). CEA repeated measures could be useful as an early surrogate marker of benefit for lung cancer patients treated with immunotherapy (26). CEA and carbohydrate antigen 153 (CA153) are common tumor biomarkers and are applied for detecting metastasis and recurrence of BC (27). CEA and CA153 are used to estimate the curative effect and detect the disease status. For example, apatinib combined with conventional chemotherapy regimens had lower CEA and CA153 levels and longer progression-free survival than conventional chemotherapy in treating patients with advanced BC (28). Meta-analysis showed that elevated CEA levels were associated with a nearly doubled risk of mortality in gastric cancer patients (29). Poor disease management increases cognitive impairment and elevates tumor biomarkers levels (30, 31). CRCI is an indicator of unsatisfactory prognosis, but its correlation with NLR, CEA, and CA153 is not clear.

In this study, we investigated the correlation between peripheral blood biomarkers (NLR, CEA, and CA153) and CRCI in 187 patients with early-stage BC before and after chemotherapy. CRCI correlation with QOL was also explored.

## Materials and methods

### Participants

We retrospectively analyzed 187 patients with early-stage BC before and after chemotherapy from August 2018 to August 2019. All patients were treated at the Affiliated Second Hospital of Anhui Medical University (Anhui, China). This study was approved by the ethics review committee of the Affiliated Second Hospital of Anhui Medical University, and all subjects provided informed consent.

Peripheral blood was obtained within the first 24 hours of admission. Patients were diagnosed with early-stage BC with immunohistochemical and imaging. Patients eligible must have had adequate cognition and activities of daily living, Eastern Cooperative Oncology Group Performance Status (ECOG PS) 0 or 1, elementary school education or above, adequate audiovisual ability to complete the questionnaire, neutrophils ≥ 1500/ul, platelets ≥ 100 × 10^3^/ul, hemoglobin concentration ≥ 8g/dl, aspartate aminotransferase and alanine aminotransferase ≤ 2.5 times the upper limit of normal (ULN), serum muscle and bilirubin ≤ 1.5 times ULN and left ventricular ejection fraction (LVEF) ≥ 50%. Patients with infectious diseases (e.g., hepatitis and syphilis), cerebral tumors, neuropathy or psychosis, advanced metastasis or cachexia, hormone or radiotherapy, major depression, anxiety, or other mental disorders, and additional impairing cognition diseases were excluded. Patients’ characteristics, blood routine, and tumor biomarker levels were extracted from the electronic patient record system.

### Neuropsychological tests

Patients received neuropsychological tests before and after chemotherapy, including mini-mental state examination (MMSE), prospective and retrospective memory questionnaire (PM and RM, PRMQ), functional assessment of cancer therapy-cognitive function version 3 (FACT-Cog, version 3, including CogPCI, CogOth, CogPCA, and CogQOL) and QOL. These questionnaires were evaluated and interpreted according to our previous study (17).

Mini-mental state examination evaluated the patients’ intellectual state and the degree of cognitive impairment comprehensively, accurately, and quickly. The MMSE total score is 30, and ≤ 26 was defined as cognitive impairment, with lower scores showing more serious (32). FACT-Cog was used to assess cognitive impairment and its impact on a patient’s quality of life *via* self-report measure. It consists of four subscales, including 37 items to evaluate the patient’s memory, concentration, attention, language, and thinking ability, and higher scores indicate more severe cognitive impairment (33). PRMQ measures prospective memory (PM) and retrospective memory (RM) lapses in daily life *via* self-reported measures. It is composed of 16 items, half about PM failures and half about RM failures, and higher scores reveal more serious memory disorders (34). QOL measures patients’ health-related quality of life. It contains 27 items and 9 attachment concerns, and higher scores indicate poorer QOL (35).

### Study procedure

We enrolled 187 patients with early-stage BC, including 66 in the before chemotherapy group (BCG) and 121 in the after chemotherapy group (ACG). All patients with ACG completed 4–6 cycles of chemotherapy based on taxane and anthracycline without intolerable side reactions. All patients received neuropsychological tests, and blood routine and tumor biomarker levels were extracted from the electronic patient record system. Patients with ACG were assigned to a cognitive normal group (CNG, *n* = 44, MMSE score > 27) and the cognitive impaired group (CIG, *n* = 77, MMSE score ≤ 26) based on the MMSE scores.

### Statistical analyses

Statistical analysis was conducted with SPSS standard version 23. The independent sample non-parametric test was used to analyze patient characteristics such as ages and years of education, NLR, CEA, and CA153 levels. The Chi-square test was used to analyze qualitative data, such as pathological patterns, tumor stage, and Her-2 expression. Questionnaires were analyzed by the independent samples *t*-test. Two-tailed *P* values of < 0.05 were considered the cutoff for statistical significance.

## Results

### Characteristics of the patients

As shown in Table 1, there was not a significant difference in age (BCG vs ACG, 51.86 ± 7.6 vs 50.554 ± 6.77, *P* = 0.348; CNG vs CIG, 50.16 ± 7.16 vs 50.78 ± 6.58, *P* = 0.752), years of education (BCG vs ACG, 8.621 ± 2.08 vs 8.82 ± 2.13, *P* = 0.58; CNG vs CIG, 8.77 ± 2.11 vs 8.84 ± 2.15, *P* = 0.87), Karnofsky performance status (KPS; BCG vs ACG, 87.12 ± 4.56 vs 87.77 ± 4.18, *P* = 0.327; CNG vs CIG, 87.5 ± 4.38 vs 87.92 ± 4.08, *P* = 0.593), tumor size (BCG vs ACG, 2.97 ± 1.4 vs 3.08 ± 1.46, *P* = 0.716; CNG vs CIG, 3.28 ± 1.33 vs 2.97 ± 1.53, *P* = 0.072), pathological pattern (BCG vs ACG, *P* = 0.943; CNG vs CIG, *P* = 0.973), tumor stage (BCG vs ACG, *P* = 0.76; CNG vs CIG, *P* = 0.978) and Her-2 expression (BCG vs ACG, *P* = 0.54; CNG vs CIG, *P* = 0.432).

**TABLE 1:** The clinical characteristics of the patients (*N* = 187).

Clinicopathological characteristics	BCG (*n* = 66)	ACG (*n* = 121)		*P*-value
			
		CNG (*n* = 44)	CIG (*n* = 77)	
Age (years)	51.9 ± 7.6	50.6 ± 6.8		0.35
		50.2 ± 7.2	50.8 ± 6.6	0.75
30–45	9 (13.6%)	7 (15.9%)	19 (24.7%)	
46–59	44 (66.7%)	33 (75.0%)	48 (62.3%)	
≥60	13 (19.7%)	4 (9.1%)	10 (13.0%)	
Education (years)	8.6 ± 2.1	8.8 ± 2.1		0.58
		8.8 ± 2.1	8.8 ± 2.2	0.87
≤6 (Primary school)	12 (18.2%)	7 (15.9%)	11 (12.3%)	
7–12 (Secondary school)	51 (77.3%)	34 (77.3%)	62 (80.5%)	
≥13 (University)	3 (4.5%)	3 (6.8%)	4 (5.2%)	
KPS	87.1 ± 4.6	87.8 ± 4.2		0.33
		87.50 ± 4.38	87.92 ± 4.08	0.59
80–89	19 (28.8%)	11 (25.0%)	15 (19.5%)	
≥90	47 (71.2%)	33 (75.0%)	62 (80.5%)	
Tumor size (cm)	3.0 ± 1.4	3.1 ± 1.5		0.72
		3.3 ± 1.3	3.0 ± 1.5	0.07
≤2	23 (34.8%)	10 (22.7%)	26 (33.8%)	
2–5	35 (53.0%)	28 (63.6%)	41 (53.2%)	
>5	8 (12.1%)	6 (13.6%)	10 (13.0%)	
**Pathological pattern (cases)**				
Infiltrative non-specific cancer	34 (51.5%)	63 (52.1%)		0.94
		23 (52.3%)	40 (51.9%)	0.97
Invasive ductal carcinoma	32 (48.5%)	58 (47.9%)		
		21 (47.7%)	37 (48.1%)	
Stage (cases)				
I	10 (15.2%)	14 (11.6%)		0.76
		5 (11.4%)	9 (11.7%)	0.98
II	32 (48.5%)	59 (48.9%)		
		21 (47.7%)	38 (49.4%)	
III	24 (36.4%)	48 (39.7%)		
		18 (40.9%)	30 (39.0%)	
Her-2				
Her-2 (+)	39 (59.1%)	77 (63.6%)		0.54
		30 68.2%)	47 (61.0%)	0.43
Her-2 (-)	27 (40.1%)	44 (36.4%)		
		14 (31.8%)	30 (39.0%)	

Data are presented as mean ± SD or n (%). SD, standard deviation; BCG, before chemotherapy group; ACG, after chemotherapy group; CNG, cognitive normal group; CIG, cognitive impairment group; KPS, Karnofsky performance status.

### Neuropsychological and quality of life assessments between groups

The cognitive function of patients decreased significantly after chemotherapy. QOL decreased significantly in patients who had completed chemotherapy or had CRCI. As shown in Table 2, the scores of MMSE (27.79 ± 1.14 vs 23.84 ± 4.11, *P* < 0.001), RM (10.32 ± 2.07 vs 15.18 ± 4.49, *P* < 0.001), PM (10.3 ± 2.01 vs 16.31 ± 5.22, *P* < 0.001), FACT-Cog (37.18 ± 3.97 vs 65.19 ± 21.67, *P* < 0.001), QOL (44.92 ± 6.27 vs 4.69 ± 22.07, *P* < 0.001) were significantly different between the BCG and ACG. Compared to the BCG, the CNG showed not a significant difference in the MMSE (27.79 ± 1.14 vs 27.93 ± 1.00, *P* > 0.05), RM (10.32 ± 2.07 vs 9.84 ± 1.83, *P* > 0.05), PM (10.3 ± 2.01 vs 10.32 ± 2.2, *P* > 0.05), FACT-Cog (37.18 ± 3.97 vs 37.86 ± 4.10, *P* > 0.05) and QOL (44.92 ± 6.27 vs 46.5 ± 5.21, *P* > 0.05) scores. In contrast, there was a statistically significant difference in the MMSE (27.79 ± 1.14 vs 21.49 ± 3.29, *P* < 0.001), RM (10.32 ± 2.07 vs 18.23 ± 1.99, *P* < 0.001), PM (10.3 ± 2.01 vs 19.74 ± 2.74, *P* < 0.001), FACT-Cog (37.18 ± 3.97 vs 80.81 ± 7.23, *P* < 0.001), and QOL (44.92 ± 6.27 vs 90.79 ± 5.59, *P* < 0.001) scores between the BCG and CIG.

**TABLE 2:** Neuropsychological tests and memory performance in the before and after chemotherapy.

Items	BCG (*n* = 66)	ACG (*n* = 121)	
		
		CNG (*n* = 44)	CIG (*n* = 77)
MMSE	27.79 ± 1.14	23.84 ± 4.11***	
		27.93 ± 1.00	21.49 ± 3.29***
RM	10.32 ± 2.07	15.18 ± 4.49***	
		9.84 ± 1.83	18.23 ± 1.99***
PM	10.30 ± 2.01	16.31 ± 5.22***	
		10.32 ± 2.20	19.74 ± 2.74***
FACT-Cog	37.18 ± 3.97	65.19 ± 21.67***	
		37.86 ± 4.10	80.81 ± 7.23***
QOL	44.92 ± 6.27	74.69 ± 22.07***	
		46.50 ± 5.21	90.79 ± 5.59***

BCG was compared with ACG, CNG, and CIG, respectively. Data are presented as the mean ± SD. SD, standard deviation; MMSE, mini-mental state examination; RM, retrospective memory; PM, prospective memory; FACT-Cog, functional assessment of cancer therapy-cognitive Function; QOL, quality of life; BCG, before chemotherapy group; ACG, after chemotherapy group. A lack of punctuation of “*” indicates that *P*-value is no significant difference. ***P < 0.001.

### Peripheral blood biomarkers and cognition

**FIGURE 1:**
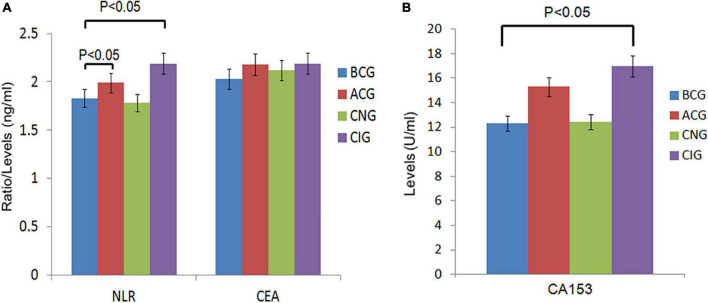
Comparison of peripheral blood biomarkers levels between groups. **(A)** Comparison of NLR and CEA levels between the 4 groups, BCG and ACG, BCG and CNG, BCG and CIG, CNG and CIG, respectively. Significant differences in NLR levels were observed between the BCG (*n* = 66) and ACG (*n* = 121), BCG (*n* = 66) and CIG (*n* = 77), *P* < 0.05. There were no significant differences in NLR levels between the BCG (*n* = 66) and CNG (*n* = 44), CNG (*n* = 44) and CIG (*n* = 77), and CEA levels, *P* > 0.05. **(B)** Comparison of CA153 levels between the 4 groups, BCG and ACG, BCG and CNG, BCG and CIG, CNG and CIG, respectively. Significant differences in CA153 levels were observed between the BCG (*n* = 66) and CIG (*n* = 77), *P* < 0.05. There were no significant differences in CA153 levels between other groups, *P* > 0.05.

The inflammatory level of patients showed an increasing trend after chemotherapy. As shown in Table 3 and Figure 1, NLR levels were significantly higher in ACG compared with BCG (*Z* = −1.996, *P* = 0.046). CEA and CA153 levels also increased in ACG compared with BCG, but did not reach statistical difference (CEA, *Z* = −0.894, *P* = 0.371; CA153, *Z* = −1.615, *P* = 0.106). As shown in Table 4, NLR, CEA and CA153 levels increased in CIG compared with CNG, but did not reach statistical difference (NLR, *Z* = −1.519, *P* = 0.129; CEA, *Z* = −0.893, *P* = 0.372; CA153, *Z* = −1.942, *P* = 0.052). As shown in Table 5 and Figure 1, NLR, CEA and CA153 levels increased in CIG compared with CNG, and NLR and CA153 levels reached statistical difference (NLR, *Z* = −2.444, *P* = 0.015; CA153, *Z* = −2.293, *P* = 0.022), and CEA levels did not reach statistical difference (*Z* = −1.195, *P* = 0.232). NLR, CEA and CA153 levels were increased in CNG compared with BCG, but there was not a significant difference (NLR, *Z* = −625, *P* = 0.532; CEA, *Z* = −0.135, *P* = 0.893; CA153, *Z* = −0.023, *P* = 0.983; respectively).

**TABLE 3:** Comparison of peripheral blood biomarkers (NLR, CEA, and CA153) levels in the BCG and ACG.

Biomarkers	BCG (*n* = 66)	ACG (*n* = 121)	*Z*	*P*-value
NLR	1.83 (1.11)	1.99 (1.48)	−1.996	0.046
CEA (ng/ml)	2.03 (1.20)	2.18 (1.68)	−0.894	0.371
CA153 (U/ml)	12.30 (9.71)	15.29 (14.77)	−1.615	0.106

Data are presented as medians (interquartile). BCG, before chemotherapy group; ACG, after chemotherapy group; NLR, neutrophil-to-lymphocyte ratio; CEA, carcinoembryonic antigen; CA153, carbohydrate antigen 153.

**TABLE 4:** Comparison of peripheral blood biomarkers (NLR, CEA, and CA153) levels in the CNG and CIG.

Biomarkers	CNG (*n* = 44)	CIG-2.293 (*n* = 77)	*Z*	*P*-value
NLR	1.78 (1.21)	2.19 (1.64)	−1.519	0.129
CEA (ng/ml)	2.12 (1.27)	2.19 (2.19)	−0.893	0.372
CA153 (U/ml)	12.43 (10.35)	16.99 (26.66)	−1.942	0.052

Data are presented as medians (interquartile). CNG, cognitive normal group; CIG, cognitive impairment group; NLR, neutrophil-to-lymphocyte ratio; CEA, carcinoembryonic antigen; CA153, carbohydrate antigen 153.

**TABLE 5:** Comparison of peripheral blood biomarkers (NLR, CEA, and CA153) levels in the BCG, CNG, and CIG.

Biomarkers	BCG (*n* = 66)	CNG (*n* = 44) CIG (*n* = 77)	*Z*	*P*-value
NLR	1.83 (1.11)	1.78 (1.21)	−0.625	0.532
		2.19 (1.64)	−2.444	0.015
CEA (ng/ml)	2.03 (1.20)	2.12 (1.27)	−0.135	0.893
		2.19 (2.19)	−1.195	0.232
CA153 (U/ml)	12.30 (9.71)	12.43 (10.35)	−0.023	0.983
		16.99 (26.66)	−2.293	0.022

Data are presented as medians (interquartile). BCG, before chemotherapy group; CNG, cognitive normal group; CIG, cognitive impairment group; NLR, neutrophil-to-lymphocyte ratio; CEA, carcinoembryonic antigen; CA153, carbohydrate antigen 153.

### Correlations

**FIGURE 2:**
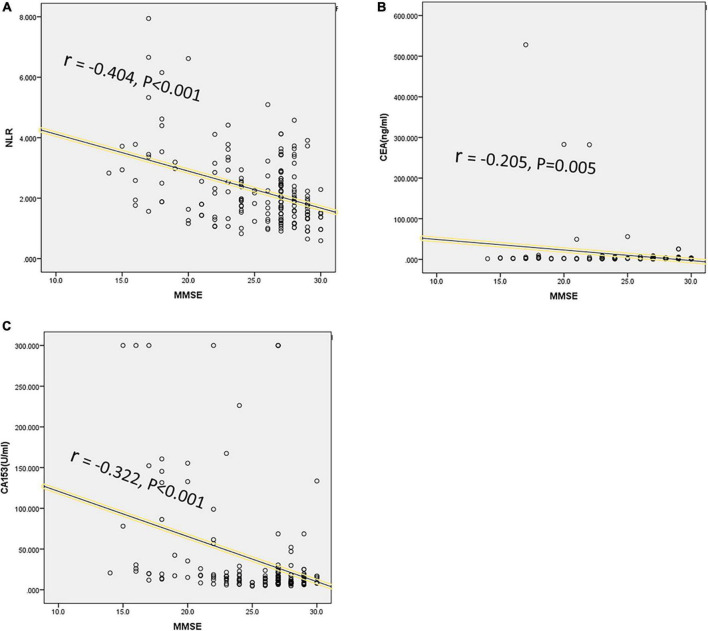
Correlations between cognition and peripheral blood biomarkers levels. The MMSE score was designed to evaluate cognition, and significant correlations were analyzed between MMSE scores and peripheral blood biomarkers levels, including NLR (*r* = -0.404, *P* < 0.001) **(A)**, CEA (*r* = -0.205, *P* = 0.005) **(B)**, and CA153 (*r* = -0.322, *P* < 0.001) **(C)**.

Cognitive function was significantly correlated with peripheral blood markers or QOL. As shown in Table 6, the scores of MMSE were significantly correlated with NLR, CEA, CA153, and QOL (*r* = −0.404, −0.205, −0.322, and −0.786; respectively, *P* < 0.001). The scores of RM, PM and FACT-Cog were significantly correlated with NLR and QOL (NLR, *r* = 0.173, 0.197, 0.214; QOL, *r* = 0.851, 0.849, 0.938; respectively, *P* < 0.05). The CogPCA scores of FACT-Cog were also significantly correlated with NLR, CEA, CA153, and QOL (*r* = 0.164, 0.16, 0.255, and 0.818; respectively, *P* < 0.05). We showed the distribution of MMSE and its correlation with NLR, CEA, and CA153 in Figure 2.

**TABLE 6:** Correlations of cognition with peripheral blood biomarkers levels and QOL.

Items	Pearson correlation coefficient			
	
	NLR	CEA	CA153	QOL
MMSE	−0.404***	−0.205**	−0.322***	0.786***
RM	0.173*	0.110	0.104	0.851***
PM	0.197**	0.140	0.143	0.849***
FACT-Cog	0.214**	0.132	0.160*	0.938***
CogPCI	0.198**	0.119	0.132	0.938***
CogOth	0.240***	0.141	0.165*	0.878***
CogPCA	0.164*	0.160*	0.255***	0.818***
CogQOL	0.186*	0.072	0.101	0.755***

Data are presented as R-values. CogPCI, CogOth, CogPCA, and CogQOL are subsets of FACT-Cog. MMSE, mini-mental state examination; RM, retrospective memory; PM, prospective memory; QOL, quality of life; FACT-Cog, functional assessment of cancer therapy-cognitive function; NLR, neutrophil-to-lymphocyte ratio; CEA, carcinoembryonic antigen; CA153, carbohydrate antigen 153. *P < 0.05; **P < 0.01; ***P < 0.001.

## Discussion

In our study, the NLR, CEA, and CA153 levels showed an increasing trend after chemotherapy, and the levels of NLR and CA153 increased significantly in patients with BC with cognitive impairment (*P* = 0.015 and 0.022, respectively). There was a strong correlation between cognitive function and QOL (*r* = −0.786, *P < 0.001*), and patients with cognitive impairment reported worse QOL. NLR was moderately correlated with cognitive function (*r* = −0.404, *P* < 0.001), while CA153 was weakly correlated (*r* = −0.322, *P* < 0.001). NLR and CA153 levels may be reliable predictive biomarkers of cognitive impairment, and cognitive impairment has a strong correlation with QOL in patients with early-stage BC.

Our study found that chemotherapy increased the level of inflammation and promoted the occurrence of CRCI. Chemotherapy is one of the important treatment methods for malignant tumors, but it also brings some negative effects to patients (36). Chemotherapy leads to cognitive impairment *via* a variety of mechanisms. For example, most commonly used chemotherapeutic drugs cannot pass through BBB, but they changed the permeability and integrity (37). Even low-dose chemotherapy caused CRCI in patients with increased BBB permeability, especially in patients with brain metastases (38, 39). DNA damage of the central nervous system induced by chemotherapy is one of the essential mechanisms of CRCI in BC (40). The gene polymorphism of apolipoprotein E (ApoE), catechol-O-methyl-transferase (COMT), and BDNF was associated with CRCI (16, 30, 41). FMRI study found that the PM impairment in patients with BC was related to the abnormality of the brain network in the hippocampus and poor connection with prefrontal brain function after chemotherapy (42). Voxel-based morphometry (VBM) measurement study found that the volume of gray matter and white matter in the prefrontal lobe, parahippocampal gyrus, thalamus, cingulate gyrus, and precuneus of patients with BC receiving chemotherapy were reduced compared with those before chemotherapy, providing direct evidence of prefrontal anatomical changes after chemotherapy (43, 44). Chemotherapeutic drugs also increased inflammatory levels and promoted the occurrence of CRCI. Our previous study found that the levels of peripheral inflammation (IL-1β, TNF-α, and IL-4) were closely related to CRCI in patients with BC (17). Zurlo et al. revealed that chemotherapy decreased NLR levels in patients with locally advanced gastric cancer and that low NLR levels were associated with a better prognosis (45). Farolfi et al. found that increased NLR levels were associated with resistance to platinum-based therapy in recurrent epithelial ovarian cancer patients (46). In our study, NLRs levels were significantly higher in patients with CRCI after chemotherapy, while slightly lower in patients without CRCI. CRCI decreases the QOL and survival time of breast cancer patients (47). The increase in NLR levels in this study confirmed this conclusion again.

Inflammation is involved in various cognitive-related diseases, including Alzheimer’s disease (AD), depression, and schizophrenia (48). Blood routine examination is widely used in the clinical evaluation of inflammatory levels, including neutrophils, monocytes, platelets, and lymphocytes. Monocyte/lymphocyte ratio (MLR), platelet/lymphocyte ratio (PLR), and NLR were often used together to evaluate inflammation levels, of which NLR was the most studied. Studies have shown that the levels of MLR, NLR, and PLR were elevated in schizophrenic patients (49). Both NLR and PLR were considered biomarkers of suicide risk in adult patients with depression, and NLR seemed to be more valuable than PLR (50). The neutrophil levels increased in AD patients, and NLR may be a diagnostic tool in AD (51). The meta-analysis also confirmed that NLR levels were elevated in patients with mood disorders compared to healthy controls (52). However, there are still no studies exploring the correlation of NLR with CRCI. Cognitive impairment is one of the symptoms of mood disorders. Consistent with previous studies, chemotherapy increased cognitive impairment and NLR levels, and patients with CRCI had higher NLR levels in our study.

The level of serum tumor markers is correlated with CRCI in patients with BC. The detection of serum tumor markers has been widely studied in the clinical stage, pathological stage, and prognosis of various cancers. The elevated level of tumor biomarkers is a manifestation of the active metabolism of cancer cells. CEA, CA125, and CA153 levels are commonly used as clinical detection indicators for BC (31). CEA and CA153 were helpful in the differential diagnosis of benign tumors and BC, but CA125 did not seem to be helpful in the detection of BC (53). Another study showed that CEA and CA153 levels increased significantly in malignant nodules compared with benign lesions, which improved the accuracy of early diagnosis and screening of BC (54). Unsatisfactory curative effect and poor prognosis of BC are high-risk factors for CRCI (55). The elevated levels of CEA and CA153 were closely related to the metastasis, progression, and poor prognosis of BC (56, 57). However, few studies have explored the correlation of CRCI between CEA and CA153. Our study showed that CRCI was weakly correlated with CA153 (*r* = −0.322, *P* < 0.001), while CEA was not (*r* = −0.205, *P* = 0.005). It may be that our sample size is small or CEA lacks specificity.

Chemotherapy-related cognitive impairment decreases the QOL of patients with BC. CRCI increased patients’ difficulty in getting along with families and integrating into society *via* impairing memory, executive function, processing speed, and attention (8). CRCI was associated with depression, anxiety, and fatigue, decreasing the QOL (58). Kiesl et al. observed that QOL enhanced significantly after CRCI improved by exercise (8). Managing Cancer and Living Meaningful (CALM) was a psychotherapy method for cancer patients (59). The QOL of patients with CRCI decreased and improved significantly after CALM intervention (60). In this study, QOL decreased significantly in patients with CRCI and was strongly correlated with CRCI (*r* = −0.786, *P* < 0.001). The results were consistent with previous studies (17, 60), but we did not conduct a CALM intervention. The CALM intervention reduced inflammatory levels (60), and future studies should explore the effects of CALM on NLR, CEA, and CA153 levels.

The limitation of this study is the single-center research, which also needs to expand the sample to verify the reliability of the conclusion. Our study only explored the correlation, and the effects of CRCI and peripheral blood biomarkers on prognosis need a long-term followed-up study. The specific mechanism of NLR and CA153 related to CRCI is still unclear, which needs to be explored in animal experiments and *in vitro* cellular experiments, such as the pathway of neutrophils promoting cognitive impairment and the effects of tumor metabolism on BBB permeability. Future studies also need to explore the effects of CALM on NLR, CEA, and CA153 levels.

## Conclusion

Our study explored the changes in easily available clinical biomarkers (NLR, CEA, and CA153) after chemotherapy and their correlation with CRCI. CRCI is a common mental disorder that should not be ignored in patients with early-stage BC, and it seriously hazards the QOL. NLR and CA153 are potentially valuable diagnostic adjuncts of CRCI. More clinical samples and long-term follow-up studies are needed to investigate the prognostic roles of NLR and CA153 in patients with CRCI.

## Data availability statement

The raw data supporting the conclusions of this article will be made available by the authors, without undue reservation.

## Ethics statement

The studies involving human participants were reviewed and approved by The Ethics Review Committee of the Affiliated Second Hospital of Anhui Medical University. The patients/participants provided their written informed consent to participate in this study.

## Author contributions

HC contributed to the conception of the study. SYu, JZ, and MW were responsible for searching the literature and writing the manuscript. GC was responsible for statistical analysis and language editing. WL, LT, and SYa contributed scientific insights and clinical data collection. LP was responsible for collecting neurological questionnaires. YJ and XY were responsible for data analysis and graphics. SYu and HC contributed to the manuscript revision and read the submitted version. All authors contributed to the article and approved the submitted version.
